# A Unique Case of a Patient With Pancreatic Cancer Developing Leptomeningeal Metastases While on Gemcitabine and Nab-Paclitaxel

**DOI:** 10.7759/cureus.58139

**Published:** 2024-04-12

**Authors:** Venkata Sireesha Chemarthi, Gunwant Guron, Hamid Shaaban

**Affiliations:** 1 Hematology and Medical Oncology, Saint Michael’s Medical Center/New York Medical College, Newark, USA; 2 Hematology and Oncology, Saint Michael’s Medical Center/New York Medical College, Newark, USA

**Keywords:** cerebrospinal fluid (csf), folfirinox chemotherapy, gemcitabine plus nab-paclitaxel therapy, pancreatic cancer, leptomeningeal carcinomatosis (lmc)

## Abstract

Brain metastases and leptomeningeal disease are rare with pancreatic cancer. Leptomeningeal disease is a catastrophic complication to have as patients deteriorate rapidly. Patients can present with symptoms of cranial nerve neuropathies, headache, nausea, and focal neurological deficits. We present a patient with metastatic pancreatic cancer who was treated initially with FOLFIRINOX (5-fluorouracil, leucovorin, irinotecan, and oxaliplatin) which resulted in marked clinical and radiologic improvement. However, he started to develop severe peripheral neuropathy and was switched to maintenance gemcitabine and nab-paclitaxel. On this regimen, his systemic disease was well controlled but he developed leptomeningeal carcinomatosis. To our knowledge, this is the first case of leptomeningeal metastases developing in a patient with pancreatic adenocarcinoma while on treatment with gemcitabine and nab-paclitaxel after cessation of FOLFIRINOX. We should maintain high clinical suspicion for leptomeningeal disease in pancreatic cancer, especially when systemic disease is well controlled, as the chemotherapeutic agents may not be crossing the blood-brain barrier effectively contributing to high morbidity and mortality.

## Introduction

Leptomeningeal disease (LMD) can be defined as metastases of cancer cells to the leptomeninges, which are thin layers of tissue covering and protecting the brain and spinal cord. Pancreatic cancer rarely metastasizes to the brain and leptomeningeal metastases are even more rare. The common solid cancers that metastasize to the leptomeninges are breast, lung, and melanoma [1]. It is a catastrophic complication to have with a poor prognosis and a median survival of less than two months without treatment [1]. Metastases to the brain from the pancreas occur in about 0.3% of the cases [2] and LMD is very rare. There are only a few cases reported in the literature of pancreatic cancer with leptomeningeal metastases [3-34]. We report a case of metastatic pancreatic cancer whose systemic disease was well controlled on chemotherapy with gemcitabine and nab-paclitaxel with very good functional status but developed LMD after which the patient deteriorated rapidly. Pancreatic cancer is still considered a relatively chemo-resistant malignancy and advancement in treatments may have led to a prolonged period of primary tumor control, but this has not seemed to be the case when it comes to the central nervous system (CNS) due to the poor penetration of chemo-agents into the cerebrospinal fluid (CSF). Our case emphasizes the highly aggressive nature of pancreatic cancer with leptomeningeal involvement and the importance of earlier identification and appropriate treatment to improve its prognosis.

## Case presentation

**Figure 1:**
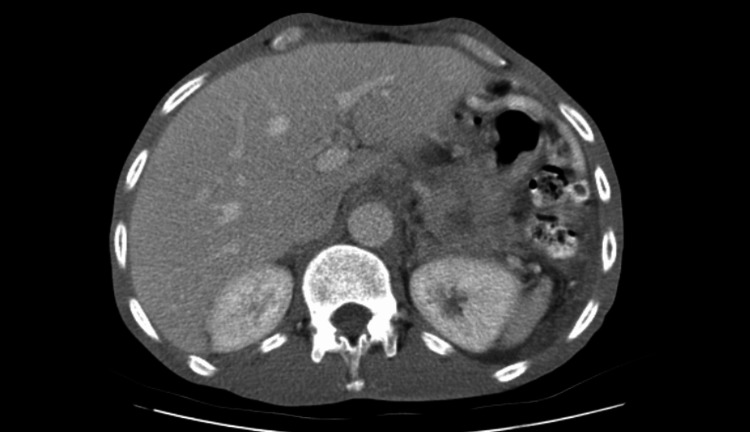
CT of the abdomen with oral and intravenous contrast. Heterogenous and hypoattenuating mass in the distal pancreas measuring 4 cm in diameter.

A 50-year-old male with no past medical history presented with complaints of one month of postprandial epigastric pain radiating to his back associated with 20-pound weight loss. He occasionally engaged in social drinking and had a history of smoking less than one pack a year which he quit 13 years ago. CT of the abdomen and pelvis with contrast revealed multiple hypoattenuating masses in the right hepatic lobe measuring up to 3 cm in diameter, concerning for metastatic disease and heterogeneous hypoattenuating mass in the distal pancreas measuring 4 cm in diameter with likely infiltration into the splenic vein causing occlusion, as seen in Figure 1. Carcinoma antigen (CA) 19-9 was elevated at 13,383 U/mL (reference range: <30 U/mL).

**Figure 2:**
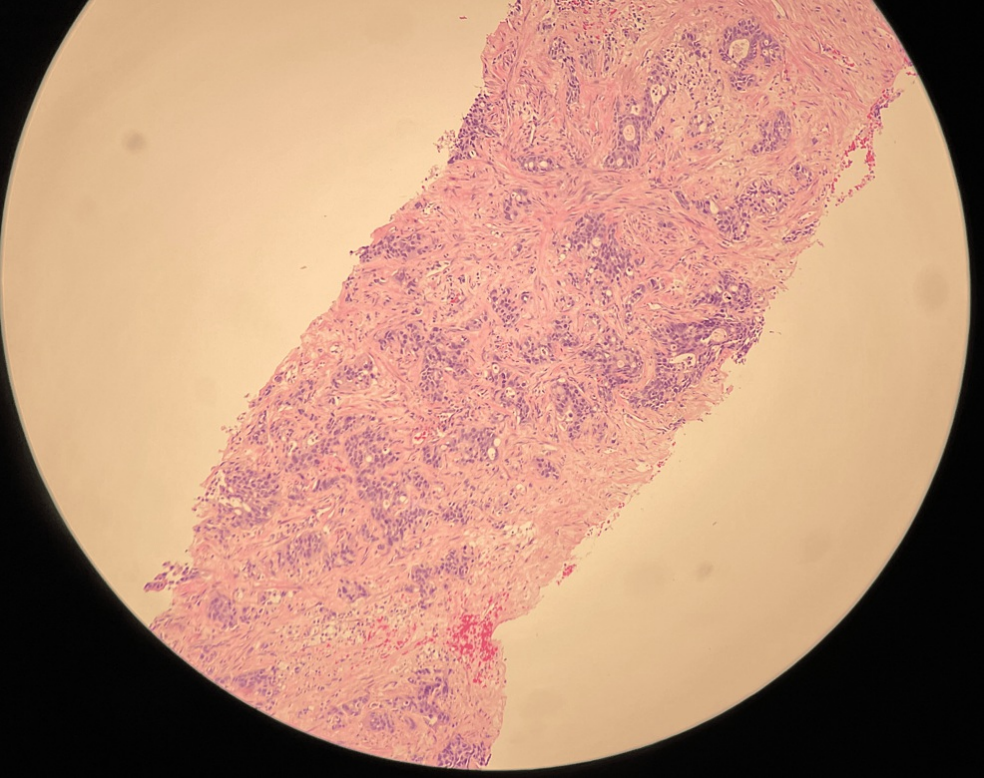
Liver mass biopsy on a low-power microscope.

**Figure 3:**
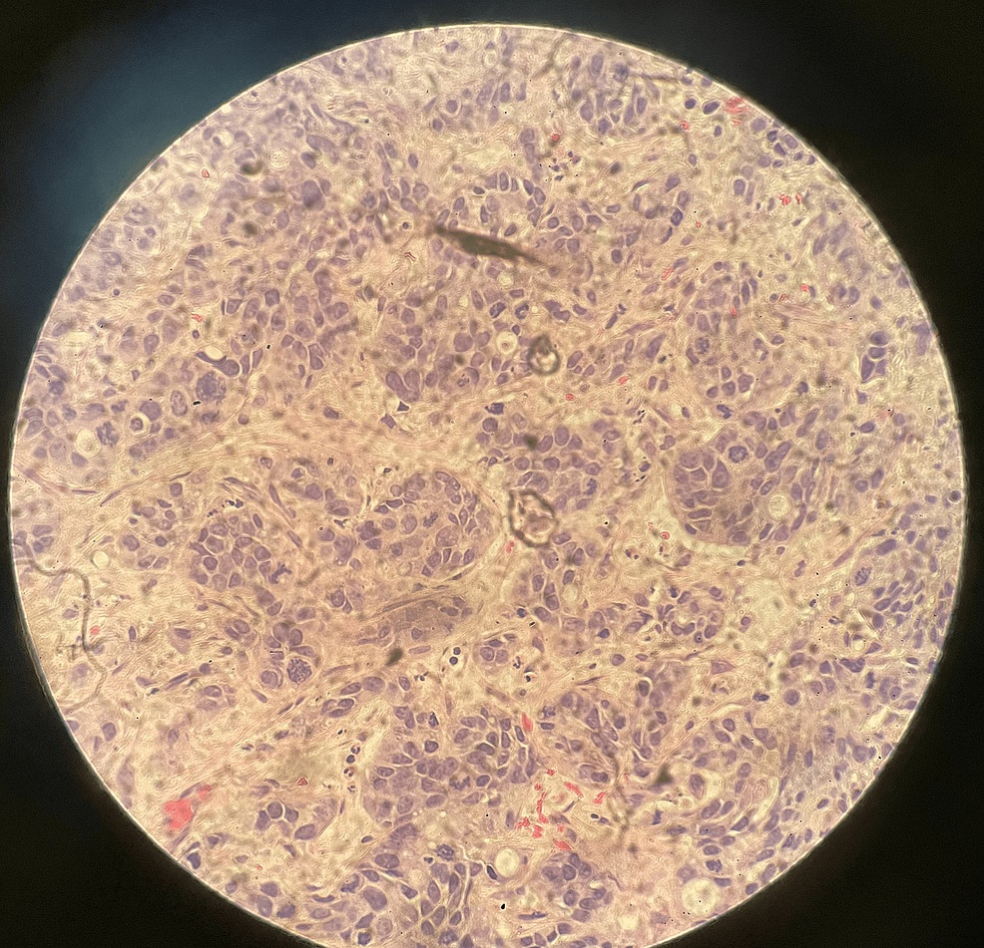
Liver mass biopsy on a high-power microscope. Infiltrating poorly differentiated large-cell carcinoma. Results of immunoperoxidase stains favor the liver lesion to be a metastatic adenocarcinoma of pancreatic origin.

An ultrasound-guided biopsy of the liver lesion revealed metastatic adenocarcinoma of pancreatic origin. Figure 2 and Figure 3 depict liver lesion biopsy findings on low-power and high-power microscopes, respectively. Immunoperoxidase stains were positive for AE1/AE3, CA 19-9, and CK7. CDX2 showed some positivity and has been reported in pancreas carcinoma but more commonly in colon or stomach adenocarcinoma. *BRCA1/2* mutations were negative. The patient received six cycles of FOLFIRINOX and the CA 19-9 decreased significantly to 274 U/mL. A repeat CT scan showed a decrease in size and conspicuity of pancreatic tail mass and significant improvement in hepatic metastases. As the patient developed neuropathy with oxaliplatin, he was switched to maintenance gemcitabine and nab-paclitaxel.

**Figure 4:**
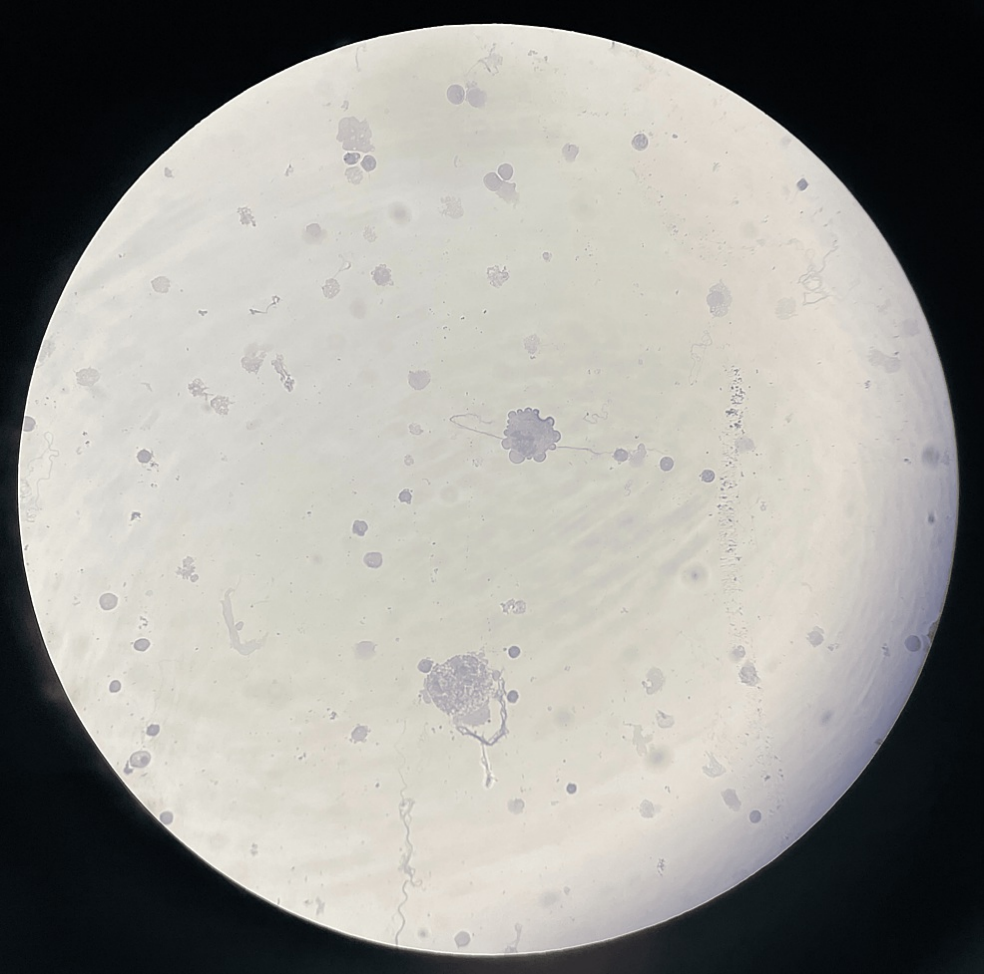
Cerebrospinal fluid cytology. Scattered few ependymal cells in a background of numerous acute inflammatory cells. Negative for malignant cells.

On his third cycle of gemcitabine and nab-paclitaxel, the patient started to complain of headaches, blurry vision, and unsteady gait for one week. He underwent an MRI of the brain in the emergency room to rule out brain metastases which showed possible leptomeningeal enhancement within the region of the cerebellum. He underwent a lumbar puncture (LP) with elevated CSF opening pressure of 26 mmHg with extremely low CSF glucose, high lymphocyte count, and protein. Gram stain and cultures were negative. CSF studies were indicative of malignancy. CSF cytology came back negative for malignant cells. We repeated LP and CSF cytology but it was negative for malignant cells; however, CSF CA 19-9 was elevated at 49 U/mL, and serum CA 19-9 increased to 449 U/mL from 245 U/mL within 10 days. Figure 4 shows CSF cytology.

During the hospital stay, the patient also experienced facial droop and ptosis indicative of facial nerve palsy. The clinical presentation, MRI imaging, and CSF studies supported the diagnosis of leptomeningeal disease due to pancreatic adenocarcinoma. He underwent repeat CT of the abdomen and pelvis which showed complete resolution of pancreatic tail mass and hepatic masses seen on prior CT scans. Radiation oncology was consulted and the patient was started on radiation therapy to the base of the skull and he also received dexamethasone therapy. We planned to give capecitabine, irinotecan, and bevacizumab in-patient chemotherapy, but he only received Irinotecan and bevacizumab as capecitabine was not available. However, the patient’s performance status deteriorated rapidly within two weeks and he was no longer a candidate for chemotherapy; hence, a referral for hospice placement was made.

## Discussion

Pancreatic cancer accounts for about 3% of all cancers in the United States and about 8% of all cancer deaths according to the Surveillance, Epidemiology, and End Results (SEER) data. Five-year relative survival is 12.5% and only 3% for distant pancreatic cancer as per SEER data. Metastases to the brain from the pancreas occur in about 0.3% of the cases [2], and LMD is very rare. Only a few case reports have been described in the literature so far. We found only around 30 cases of pancreatic cancer with LMD reported in the literature so far (PubMed, EMBASE, Ovid, and Google Scholar) [3-34].

Patients with LMD present with symptoms of cranial neuropathies, focal motor deficits, headache, nausea, and focal neurological deficits that are pertinent to the location of the involved leptomeninges [1]. The symptoms occur due to malignant cells blocking CSF drainage/infiltrating the cranial nerves [1]. Our patient presented with a headache and unsteady gait reflecting cerebellar involvement, and an MRI of the brain confirmed leptomeningeal enhancement of the cerebellum. Our patient also had facial nerve palsy which improved with the administration of dexamethasone.

The definitive diagnosis of LMD is by detection of malignant cells on CSF cytology; however, multiple LPs may be required to establish positive CSF cytology as initial CSF is positive in only about 50% of the patients [35]. In our patient, we repeated LP twice but the CSF cytology was negative for malignant cells. However, the MRI brain findings combined with elevated tumor marker CA 19-9 of 49 U/mL in the CSF and very low glucose, high protein, and lymphocytes with negative gram stain and bacterial and fungal cultures confirmed the diagnosis of leptomeningeal metastases. No studies have determined the optimal treatment regimen for patients with pancreatic cancer and LMD, given the rarity. The data for treatment outcomes is mainly from single case reports described in the literature. Our patient’s systemic disease was very well controlled after six cycles of FOLFIRINOX, as demonstrated by the resolution of abdominal disease on the CT scans. He had no complaints other than severe peripheral neuropathy related to oxaliplatin. It is very important to note that this regimen contained irinotecan which has good CNS penetration. The decision was made to switch to gemcitabine and nab-paclitaxel to avoid worsening the patient’s neuropathy. We achieved control of abdominal disease; however, he progressed with LMD.

In the case report described by Hirota et al. [10], the patient was treated with gemcitabine and whole-brain radiation therapy and lived 3.5 years after the diagnosis of LMD was made. This case has the longest survival reported so far. Iwatsuka et al. [23] treated the patient with nab-paclitaxel plus gemcitabine and the patient lived for almost six months. Ceccon et al. [24] reported a patient treated with gemcitabine plus nab-paclitaxel who survived for three months. In another case report by Johnson et al. [14], the patient survived nine months after the diagnosis of LMD and he was treated with capecitabine and irinotecan, intrathecal topotecan, and bevacizumab, along with total brain irradiation.

Unfortunately, our patient developed LMD while on gemcitabine and nab-paclitaxel with rapid progression in contrast to what was observed in the previous case reports [10,23,24], which suggests that this regimen does not ideally penetrate the blood-brain barrier very well. The longer survival benefit seen in these cases [10,14] might have been due to total brain irradiation.

Along with local radiation to the cerebellum, we planned to administer capecitabine, irinotecan, and bevacizumab in-patient chemotherapy as there is evidence that these drugs cause blood-brain barrier [36,37], however, he only received irinotecan and bevacizumab as capecitabine was not available. The patient’s performance status deteriorated rapidly within two weeks of the diagnosis of LMD making him a poor candidate for further therapy.

The majority of the patients described in the literature received palliative care and few patients received radiation therapy alone. Table 1 summarizes case reports of patients who received treatment for LMD with chemotherapy, radiotherapy, or both.

**Table 1:** Summary of pancreatic cancer patients with leptomeningeal disease who received treatment. LMD = leptomeningeal disease

Authors	Age in years and gender	Chemotherapy (after diagnosis of LMD)	Radiation therapy	Time to LMD diagnosis	Survival (after LMD diagnosis)
Hirota et al., 2008 [10]	64, Male	Gemcitabine	Yes (TBI)	2 months	3.5 years
Johnson et al., 2018 [14]	53, Male	Capectabine, irinotecan, bevacizumab, and intrathecal topotecan	Yes (TBI)	29 months	9 months
Rebischung et al., 2008 [25]	44, Female	Intrathecal methotrexate and 125 IUdR	No	34 months	6 months
Iwatsuka et al., 2021 [23]	57, Female	Nab-paclitaxel plus gemcitabine	Yes	0	5.75 months
Amico et al., 2016 [27]	57, Male	No	Yes	11 months	4 months
Ceccon et al., 2020 [24]	51, Male	Nab-paclitaxel plus gemcitabine	No	30 months	3 months
Farahvash et al., 2020 [28]	69, Female	No	Yes	9 years	3 months
Ferreira et al., 2001 [7]	49, Male	Intrathecal methotrexate, cytarabine, and thiotepa	No	4 months	2 months
Hong et al., 2014 [6]	72, Female	Carboplatin, paclitaxel, and palareorep	No	7 months	2 months
Yim et al., 2022 [20]	58, Female	No	Yes	10 months	2 months
Minchom et al., 2010 [11]	59, Male	Intrathecal methotrexate, dexamethasone, cytarabine, and systemic gemcitabine	No	0	1.5 months
O’Connor et al., 2021 [19]	65, Male	No	Yes	12 months	1.1 months
Assaf et al., 2021 [18]	48, Male	No	Yes	12 months	1 month
Sayanagi et al., 2022 [22]	49, Female	Intrathecal methotrexate	No	3 years	Many days
Amico et al., 2016 [27]	42, Female	No	Yes	18 months	Few weeks
Yoo et al, 2015 [4]	80, Male	No	Yes	0	Not reported
Rao et al, 2013 [13]	57, Male	FOLFIRINOX	Yes	0	Not reported

## Conclusions

Leptomeningeal metastases from pancreatic adenocarcinoma are very rare. It is important to maintain high clinical suspicion and be aware of the presentation of LMD in pancreatic cancer, especially when the systemic disease is controlled, as seen in our case. The incidence of LMD may be increasing in pancreatic cancer with the systemic disease being controlled and patients living longer. The chemotherapy agents effective in controlling the systemic disease may not be crossing the blood-brain barrier effectively contributing to rapid progression and mortality. Whole-brain radiation therapy may be beneficial, as seen in the two case reports; however, there are no studies for optimal management of pancreatic cancer with leptomeningeal metastases. Larger studies are needed to establish this if the incidence continues to increase.

## References

[1] Le Rhun E, Taillibert S, Chamberlain MC (2013). Carcinomatous meningitis: leptomeningeal metastases in solid tumors. Surg Neurol Int.

[2] Park KS, Kim M, Park SH, Lee KW (2003). Nervous system involvement by pancreatic cancer. J Neurooncol.

[3] Anne M, Ahmad N, Lee P, Aziz M, Lebowicz Y (2013). An unusual presentation of isolated leptomeningeal disease in carcinoma of unknown primary with pancreatic features. J Investig Med High Impact Case Rep.

[4] Yoo IK, Lee HS, Kim CD (2015). Rare case of pancreatic cancer with leptomeningeal carcinomatosis. World J Gastroenterol.

[5] Trinh VT, Medina-Flores R, Chohan MO (2016). Leptomeningeal carcinomatosis as primary manifestation of pancreatic cancer. J Clin Neurosci.

[6] Hong CS, Kurt H, Elder JB (2014). Asynchronous leptomeningeal carcinomatosis from pancreatic cancer: a case report and review of the literature. Clin J Gastroenterol.

[7] Ferreira Filho AF, Cardoso F, Di Leo A, Awada A, da Silva VD, Tovar RB, Schwartsmann G (2001). Carcinomatous meningitis as a clinical manifestation of pancreatic carcinoma. Ann Oncol.

[8] Kurzaj E, Kopczynski S, Barowska-Lehman J, Ludwiczak R (1980). Subdural haematoma associated with dural carcinomatosis in a patient with primary carcinoma of pancreas. Neurochirurgia (Stuttg).

[9] Yagi Y, Nishimura Y, Nakatsugawa S, Fukuoka T, Hirota M, Okamoto K, Sato T, Ichihara T (2006). A case of meningeal carcinomatosis from pancreatic cancer during chemotherapy using gemcitabine. Jpn J Gastoenterol Surg.

[10] Hirota M, Yagi Y, Yamashita K, Okamoto K, Sato T, Ichihara T (2008). [A long survival case of unresectable pancreatic cancer by chemoradiotherapy with gemcitabine as key drug]. Gan To Kagaku Ryoho.

[11] Minchom A, Chan S, Melia W, Shah R (2010). An unusual case of pancreatic cancer with leptomeningeal infiltration. J Gastrointest Cancer.

[12] Blows SJ, Morgan R, Dhariwal U, Petts G, Roncaroli F (2012). Pancreatic adenocarcinoma presenting with sudden onset bilateral deafness secondary to metastatic leptomeningeal infiltration. Age Ageing.

[13] Rao R, Sadashiv SK, Goday S, Monga D (2013). An extremely rare case of pancreatic cancer presenting with leptomeningeal carcinomatosis and synchronous intraparenchymal brain metastasis. Gastrointest Cancer Res.

[14] Johnson WR, Theeler BJ, Van Echo D, Young P, Kwok M (2018). Treatment of leptomeningeal carcinomatosis in a patient with metastatic pancreatic cancer: a case report and review of the literature. Case Rep Oncol.

[15] Ikeda Y, Yoshida M, Ishikawa K (2020). Pancreatic cancer with leptomeningeal carcinomatosis: case report and literature review. Int Cancer Conf J.

[16] Grira MT, Ben Jemaa HM, Lammouchi TM, Benammou SA (2007). Meningitis revealing pancreatic carcinoma. Neurosciences (Riyadh).

[17] Naqvi SA, Ahmed I (2015). Carcinomatous meningitis: a rare complication of pancreatic adenocarcinoma. J Coll Physicians Surg Pak.

[18] Assaf I, Mans L, Sakr R, Verset G, Van Laethem JL (2021). Unusual metastasis in BRCA mutated pancreatic cancer while on maintenance Olaparib: two case reports and review of the literature. Eur J Cancer.

[19] O'Connor CA, Park JS, Kaley T (2021). Leptomeningeal disease in pancreas ductal adenocarcinoma: a manifestation of longevity. Pancreatology.

[20] Yim E, Leung D (2022). Leptomeningeal disease in BRIP1-mutated pancreatic adenocarcinoma. BMJ Case Rep.

[21] Walker DD, Kubas BG, Clifton GT, Burris JK, Rendo MJ (2023). Leptomeningeal carcinomatosis in a patient with PALB2-mutated pancreatic adenocarcinoma: a case report and review of the literature. Curr Prob Cancer Case Rep.

[22] Sayanagi T, Ohishi Y, Katayama M, Tamura R (2022). Leptomeningeal carcinomatosis in a patient with pancreatic cancer: a rare phenomenon?. Medicines (Basel).

[23] Iwatsuka K, Kikuta D, Shibuya H (2021). Treatment outcome of nab-paclitaxel plus gemcitabine for leptomeningeal carcinomatosis from pancreatic ductal adenocarcinoma: an autopsy case report. Intern Med.

[24] Ceccon G, Wollring M, Brunn A, Deckert M, Waldschmidt D, Fink GR, Galldiks N (2020). Leptomeningeal carcinomatosis in a patient with pancreatic cancer responding to nab-paclitaxel plus gemcitabine. Case Rep Oncol.

[25] Rebischung C, Hoffmann D, Stefani L (2008). First human treatment of resistant neoplastic meningitis by intrathecal administration of MTX plus (125)IUdR. Int J Radiat Biol.

[26] Galatioto S, Savettieri G (1975). [Meningeal carcinomatosis secondary to a primary pancreatic tumor. Anatoma-clinical study]. Acta Neurol (Napoli).

[27] Amico AL, Lukas RV, Kindler HL (2016). Leptomeningeal carcinomatosis from pancreatic cancer associated with germline BRCA mutations: case reports and review of the literature. J Pancreas.

[28] Farahvash A, Knox JJ, Micieli JA (2020). Severe optic neuropathy as the presenting sign of leptomeningeal carcinomatosis from pancreatic cancer. Can J Ophthalmol.

[29] Yarra P, Thakur K, Tripathi N, Aknabi O, Kudaravalli P (2019). Metastatic pancreatic cancer in 19-year-old. J Gastroenterol.

[30] Perilla AS, Melian E, Schneck M (2017). Pancreatic cancer with leptomeningeal carcinomatosis: case presentation and literature review. Neurology.

[31] Na BS, Song SJ, Song JM, Woo HG, Kwon YN, Lee D, Ahn TB (2015). Stroke-like manifestation in a patient with leptomeningeal metastasis of pancreatic cancer. J Dig Cancer Res.

[32] Jindal S, Adithya GK, Madaan V, Gupta R, Tandon V, Govil D (2018). A rare case of carcinoma pancreas with meningeal metastasis. Apollo Med.

[33] Bain E, Cheon P, Wong E (2015). Pancreatic cancer with rare leptomeningeal disease: a case report and literature review. J Pain Manage.

[34] Slostad JA, Hallemeier CL, Bamlet WR, Couch F, McWilliams RR (2019). Leptomeningeal carcinomatosis in BRCA-mutated pancreatic cancer. J Clin Oncol.

[35] Kesari S, Batchelor TT (2003). Leptomeningeal metastases. Neurol Clin.

[36] Rivera E, Meyers C, Groves M (2006). Phase I study of capecitabine in combination with temozolomide in the treatment of patients with brain metastases from breast carcinoma. Cancer.

[37] Friedman HS, Prados MD, Wen PY (2009). Bevacizumab alone and in combination with irinotecan in recurrent glioblastoma. J Clin Oncol.

